# Outer Membrane Protein of Gut Commensal Microorganism Induces Autoantibody Production and Extra-Intestinal Gland Inflammation in Mice

**DOI:** 10.3390/ijms19103241

**Published:** 2018-10-19

**Authors:** Naoko Yanagisawa, Hidehiro Ueshiba, Yoshihiro Abe, Hidehito Kato, Tomoaki Higuchi, Junji Yagi

**Affiliations:** Microbiology and Immunology, Tokyo Women’s Medical University, Tokyo 162-8666, Japan; ueshiba.hidehiro@twmu.ac.jp (H.U.); lagwagon0423@yahoo.co.jp (Y.A.); kato.hidehito@twmu.ac.jp (H.K.); higuchi.tomoaki@twmu.ac.jp (T.H.); yagi.junji@twmu.ac.jp (J.Y.)

**Keywords:** commensal bacteria, autoantibody, Harderian gland, salivary gland

## Abstract

Gut commensal microorganisms have been linked with chronic inflammation at the extra-intestinal niche of the body. The object of the study was to investigate on the chronic effects of a gut commensal *Escherichia coli* on extra-intestinal glands. The presence of autoimmune response was diagnosed by autoantibody levels and histological methods. Repeated injection of *E. coli* induced mononuclear cell inflammation in the Harderian and submandibular salivary glands of female C57BL/6 mice. Inflammation was reproduced by adoptive transfer of splenocytes to immune-deficient Rag2 knockout mice and CD4^+^ T cells to mature T cell-deficient TCRβ-TCRδ knockout mice. MALDI TOF mass spectrometry of the protein to which sera of *E. coli*-treated mice reacted was determined as the outer membrane protein A (OmpA) of *E. coli*. Multiple genera of the Enterobacteriaceae possessed OmpA with high amino-acid sequence similarities. Repeated injection of recombinant OmpA reproduced mononuclear cell inflammation of the Harderian and salivary glands in mice and elevation of autoantibodies against Sjögren’s-syndrome-related antigens SSA/Ro and SSB/La. The results indicated the possibility of chronic stimuli from commensal bacteria-originated components as a pathogenic factor to elicit extra-intestinal autoimmunity.

## 1. Introduction

The structure of the human gut microbiota is established early in life, and thereafter maintains a symbiotic relationship with their host in an anaerobic environment for decades [1,2]. *Escherichia coli*, a facultative anaerobe from the Proteobacteria phylum and a key member of the Enterobacteriaceae family [3], colonize the intestine of human infants within hours of birth [4], by their ability to respire oxygen in the intestine of newborns [5,6]. Although early colonization of *E. coli* is indispensable for the development of oral tolerance [7], Enterobacteriaceae constitute only a small fraction of less than 1% of the gut microbiota in healthy adults [8] due to restricted carbohydrate sources that they need for growth, for which they compete with the obligate anaerobe counterparts [9]. Nitrate can be used for anaerobic respiration in Enterobacteriaceae, thus conferring growth advantage to *E. coli* by a high protein diet lifestyle [10,11,12,13]. Dysbiosis of increased facultative anaerobes exceeds commensal tolerance and stimulates innate immune activation beyond the intestinal niche [14], such as atherosclerosis [14,15]. Several of the bacterial components have been implicated in the development of autoimmunity, including the microbial-von Willebrand factor type A domain protein (vWFA) [16] and the flagellin FliC [17] in sialoadenitis of Sjögren’s syndrome (SS), and the outer membrane protein OmpC in the synovial membrane of rheumatoid arthritis [18].

OmpA is one of the most abundant proteins expressed at high levels up to 100,000 copies per cell on the outer membrane of Enterobacteriaceae [19] to maintain cell integrity under osmotic stress [20]. The structure of OmpA from *E. coli* is composed of 325 amino acids consisting of N-terminal 171 residues, which adopt β-barrel domain composed of eight membrane-spanning β-strands [21]. An alternative temperature dependent conformation consists of 16-stranded β-barrel involving an additional eight β-strands from the C-terminal domain, which unless they reside in the periplasm, corresponding to a large pore [22]. Given its copy number and location, OmpA also interacts with and modulates host immunity by inactivating the complement cascade [20,23], and evading leukocyte killing by suppression of reactive oxygen species [24]. Therefore, attention has been paid to OmpA as a multifunctional immune modulating enterobacterial protein [25].

The present study was objected to investigate the possibility of gut microorganism in the pathogenesis of extra-intestinal autoimmunity. The presence of autoimmunity in the present study was diagnosed in female C57BL/6 mice by autoantibody levels and histology of the head and neck glands, including the submandibular salivary gland and the retro-orbital Harderian gland, which possesses combined species-variable abilities in exocytotic lipid-rich secretion, melatonin and porphyrin synthesis [26], and retinoic acid biosynthesis as the source of molybdoflavoenzyme [27]. The findings of the present study indicate that chronic stimuli from the outer membrane protein of commensal *E. coli* may act to trigger inflammatory cell infiltration in the extra-intestinal glands and production of autoantibodies.

## 2. Results

### 2.1. Effect on Extra-Intestinal Gland Inflammation by Systemic Treatment of Mice with Bacterial Components

**Figure 1:**
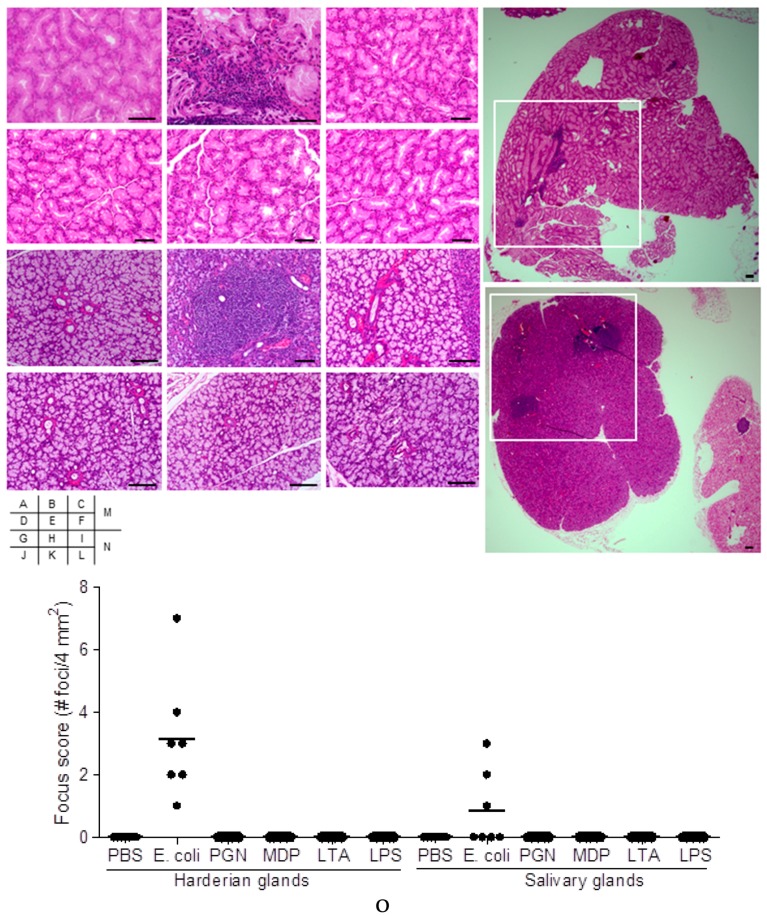
Histology of mice injected with bacteria or bacterial cell wall components. C57BL/6 female mice were repeatedly treated with *E. coli* (**B**,**H**,**M**,**N**), PGN (**C**,**I**), MDP (**D**,**J**), LTA (**E**,**K**) or LPS (**F**,**L**), respectively, for eight weeks. Harderian glands (**B**–**F**,**M**) and salivary glands (**H**–**L**, **N**) fifteen weeks after the final inoculation of *E. coli* were visualized by hematoxylin and eosin (HE) staining and were compared to those of mice treated with PBS alone (**A**,**G**, respectively). A representative inflammatory focus in one mouse of seven mice per treatment group is presented (**A**–**L**). A representative area of 2 mm × 2 mm is indicated as a white-lined square in a histologic section of the Harderian gland (**M**) and the salivary gland (**N**), respectively, visualized at low magnification (4×). Scale bar, 100 μm. Quantification of inflammatory foci in Harderian and salivary glands. The number of inflammatory foci, each containing at least 50 mononuclear cells, was defined as # foci (**O**). Data presented are individual values (*n* = 7) plotted with a bar indicating the group mean. Differences between the groups of measurements were analyzed by Kruskal-Wallis with post-test comparison. Focus scores of the Harderian glands in *E. coli*-treated group were significantly higher (*p* < 0.05) compared to all other measurement groups (**O**). PBS: phosphate buffered saline; PGN: peptidoglycan; MDP: muramyl dipeptide; LTA: lipoteichoic acid; LPS: lipopolysaccharide.

To examine whether chronic exposure of bacteria or bacterial cell wall components could induce autoimmunity in mice by systemic immunization, six weeks old mice were treated with *E. coli*, peptidoglycan (PGN), muramyl dipeptide (MDP), lipoteichoic acid (LTA), lipopolysaccharide (LPS) or phosphate buffered saline (PBS), by intraperitoneal injection once a week for a total of eight weeks. Harderian glands and salivary glands were histologically examined fifteen weeks after the final treatment. Harderian glands of *E. coli*-treated mice showed infiltration of inflammatory cells (Figure 1B,M), in comparison to no inflammation in those of mice treated with PBS alone (Figure 1A). The incidence of at least one foci of inflammation in the Harderian gland occurred in all seven mice of the *E. coli*-treated group (Figure 1O). Inflammation in the Harderian gland was not detected in mice receiving PGN, MDP, LTA or LPS (Figure 1C–F). Inflammatory cells were present in the salivary glands of *E. coli*-treated mice (Figure 1H,N). Incidence of at least one foci in the salivary gland occurred in three of a total of seven mice in the *E. coli*-treated group (Figure 1O). Inflammation in the salivary gland was not detected in mice treated with PGN, MDP, LTA, LPS, or PBS (Figure 1G,I–L). Quantification of the inflammatory foci showed that foci were scattered within one sample organ (Figure 1O).

### 2.2. Cell-Mediated Immunity in Bacteria-Treated Mice

**Figure 2:**
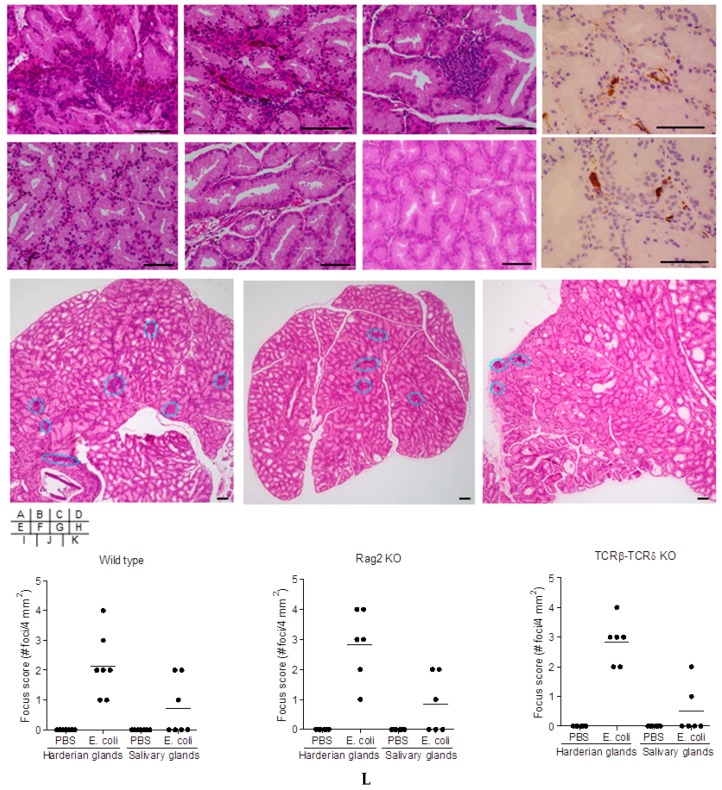
Histology of mice injected with *E. coli*. Harderian glands of *E. coli*-treated (**A**,**D**,**H**,**I**) and PBS-treated (**E**) C57BL/6 wild type mice were histologically examined fifteen weeks after the final injection. Harderian glands of Rag2 KO mice (**B**,**F**,**J**) and TCRβ-TCRδ KO mice (**C**,**G**,**K**) were histologically examined one week after adoptive transfer of splenocytes (**B**,**F**,**J**) or CD4^+^ T cells (**C**,**G**,**K**) from *E. coli*-inoculated (**B**,**C**,**J**,**K**) or PBS-inoculated (**F**,**G**) C57BL/6 donor mice. Inflammatory infiltrates were examined by HE-staining (**A**–**C**,**E**–**G**,**I**–**K**) and immune-staining for CD3 (**D**) and IgG1 positive cells (**H**). A representative inflammatory focus in one mouse of six to seven mice per treatment group is presented. Inflammatory foci are indicated inside dotted lines in a representative 4 mm^2^ histology section of the Harderian gland of one mouse from each treatment group (**I**–**K**). Scale bar, 100 µm. Quantification of inflammatory foci in the Harderian and salivary glands of wild type (*n* = 7), Rag2 KO (*n* = 6), and TCRβ-TCRδ KO (*n* = 6) mice (**L**). Data presented are individual values plotted with a bar indicating the group mean. Differences between the groups of measurements were analyzed by Kruskal-Wallis with post-test comparison. Focus scores of the Harderian glands in *E. coli*-treated group were significantly higher (*p* < 0.05) compared to all other measurement groups (**L**).

*E. coli* induced accumulation of inflammatory cells in the Harderian glands (Figure 2A,I,L). No inflammatory cells were observed in mice treated with PBS alone (Figure 2E,L). Infiltrating cells in the Harderian glands of the *E. coli*-treated mice were in part CD3 positive cells detected by immunohistochemistry (Figure 2D), which were not detected in the PBS-treated mice. IgG1 positive cells were scattered within the stromal tissue of the Harderian glands of mice by *E. coli* treatment (Figure 2H). IgG1 cells were not observed in the Harderian glands of mice treated with PBS.

Adoptive transfer experiments were undertaken to elucidate the role of cell-mediated immune responses in mice induced by repeated *E. coli* injection. Adoptive transfer of bulk splenocytes to Rag2 KO mice from *E. coli*-treated donor mice developed accumulation of inflammatory cells in the Harderian glands (Figure 2B,J,L), compared to those from donor mice receiving PBS alone (Figure 2F,L). Similarly, Harderian glands of TCRβ-TCRδ KO mice subsequent to adoptive transfer of spleen-derived CD4^+^ T cells from *E. coli*-treated donor mice showed infiltration of inflammatory cells (Figure 2C,K,L), but not those from donor mice receiving PBS alone (Figure 2G).

### 2.3. Identification of OmpA from E. coli as a Representative Immunogenic Protein in Mice

**Figure 3:**
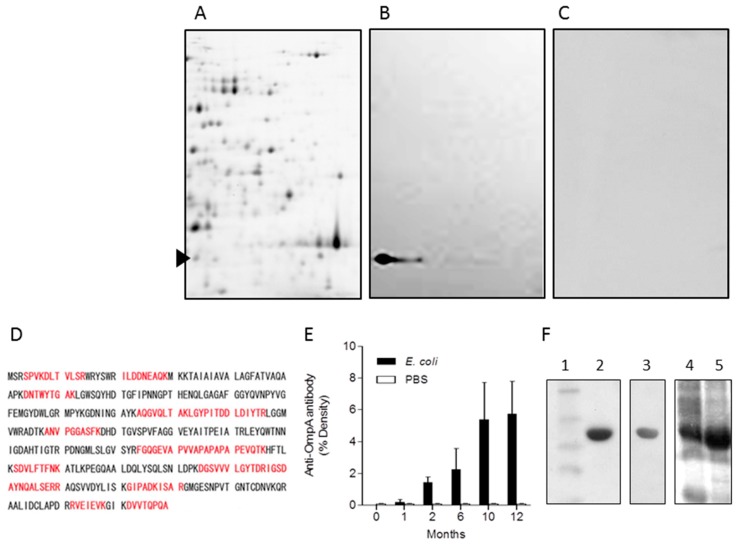
Immunogenic protein in *E. coli*-treated mouse model. Cell surface proteins of *E. coli* were separated on 2-D PAGE (**A**). Western blotting of 2-D gel-transferred nitrocellulose membrane was probed with serum of *E. coli*-treated mouse (**B**), or serum of mouse treated with PBS alone (**C**). Representative membranes of similar Western blotting results with sera of three sets of mice are shown. A spot of 41 kDa/pI 6.24 in *E. coli*-surface extract reacting with sera from *E. coli*-treated mouse was excised from the 2-D gel (arrow head in A). OmpA was identified by MALDI-TOF/MS using peptide mass fingerprinting (Matrix Science, London, UK) and the non-redundant National Center for Biotechnology Information (NCBI) database (US National Library of Medicine, Bethesda, MD, USA) with a Mascot search engine (Matrix Science) through the BioTools 3.0 interface (Bruker Daltonics, Billerica, MA, USA). Matched peptides from the MS-fit data are shown in bold red (**D**). Anti-OmpA antibodies in sera of mice were compared between those of *E. coli*-treated mice (*n* = 5), and those of PBS-treated mice (*n* = 5) at indicated time after the final injection (*p* = 0.02 at 1 month, *p* < 0.01 at 2, 6, 10, and 12 months, Mann–Whitney *U* test) (**E**). Recombinant OmpA was expressed and purified (**F**). Molecular weight markers indicate 58, 46, 30, 25, and 17 kDa, respectively (lane 1). Ni^2+^-affinity column-purified hexahistidine (6×His)-tagged-OmpA (Lane2). Western blotting of whole cell lysate of BL21 (DE3) transformed with pQE2::*ompA* probed with anti-OmpA monoclonal antibody clone 49.4-15 [28] (lane 3). SDS-PAGE of whole cell lysate of BL21 (DE3) pQE2::*ompA* before (lane 4) and after isopropyl β-d-1-thiogalactopyranoside (IPTG) induction (lane 5).

We sought to identify the cellular component of *E. coli* that contributes to the generation of autoimmunity. Cell surface extracts of *E. coli* separated on 2-D gels showed numerous numbers of proteins at molecular weights between 25–100 kDa and pI 6–11 (Figure 3A). Western blotting of 2-D PAGE gel-transferred nitrocellulose membrane probed with sera of the experimental dacryoadenitis mice showed an immune-reactive protein migrating at 41 kDa/pI 6.24 (Figure 3B), which was not detected with sera of mice inoculated with PBS alone (Figure 3C). Analysis of the 41 kDa/pI 6.24 spot excised from the 2-D PAGE-gel of *E. coli* surface fraction by MALDI-TOF mass spectrometry was identified as OmpA (theoretical Mw/pI of 35,081.25/5.42), with MS-fit sequence coverage of 38% (Figure 3D). To elucidate the immunogenicity of OmpA in vivo, sera of mice treated once a week during eight weeks with *E. coli* was validated by the production of antibodies against OmpA. Serum anti-OmpA antibody titer levels were elevated by systemic *E. coli* injection and were increased over time up to 10 months after completion of the final injection of *E. coli* (Figure 3E).

### 2.4. Analysis of the Amino Acid Sequences of OmpA from Phylum Proteobacteria

**Figure 4:**
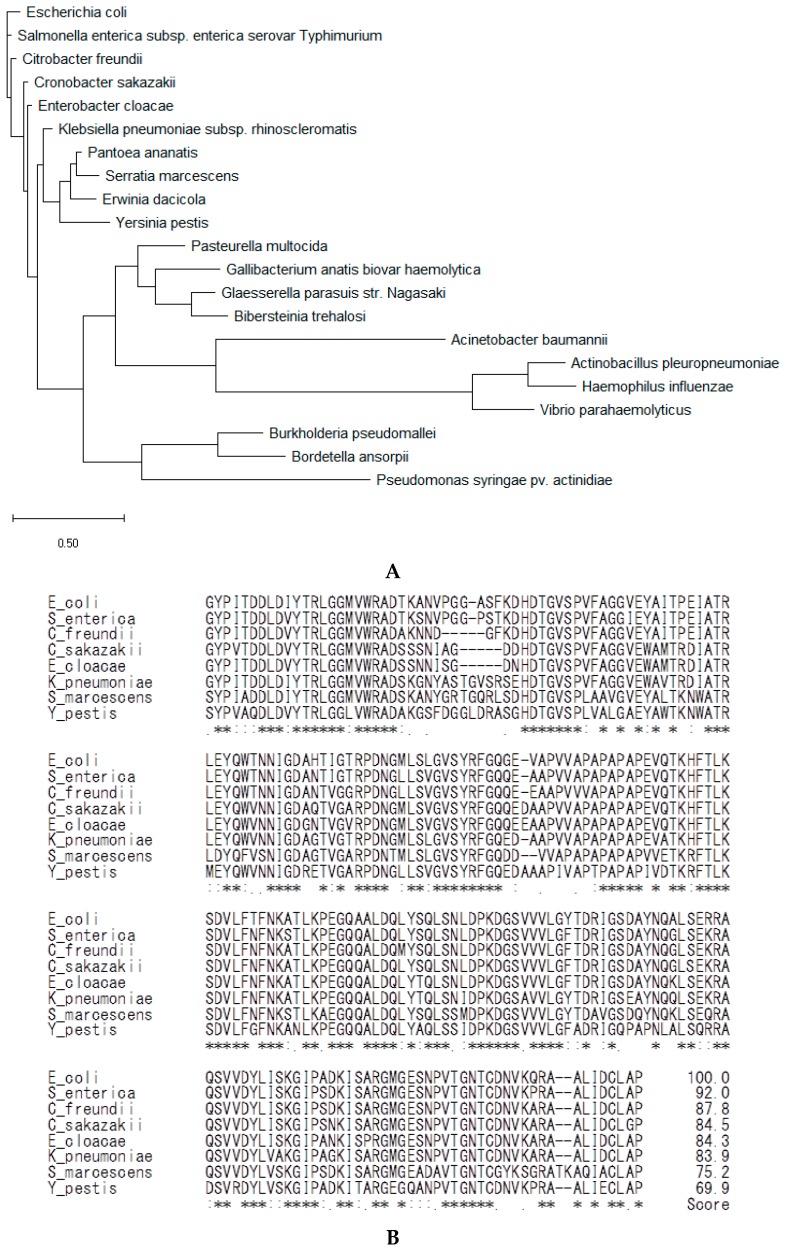
Phylogenetic analysis of the Proteobacteria pylum based on OmpA amino acid sequences by Maximum Likelihood method conducted on MEGA X software (Molecular Evolutionary Genetics Analysis, State College, PA, USA), as described in Reference [29]. The tree is drawn to scale, with branch lengths measured in the number of substitutions per site. Genera of Proteobacteria phylum included those of classes Gammaproteobacteria and Betaproteobacteria (**A**). Multiple sequence alignment of OmpA G80 to P305 of *E. coli* ATCC 25922 with amino acids of Gammaproteobacteria/Enterobacteriales by ClustalW (Available online: https://www.genome.jp/tools-bin/clustalw). Conserved residues are highlighted with asterisks. Amino acids exhibiting strong and weak similarities according to point-accepted-mutation are indicated by colons and points, respectively. Scores (%) of aligned similarities compared with those of *E. coli* ATCC 25922 are shown (**B**).

Phylogenetic analysis of OmpA from phylum Proteobacteria showed that the bacteria from ten genera of the Enterobacteriaceae family (*Escherichia*, *Salmonella*, *Citrobacter*, *Cronobacter, Enterobacter*, *Klebsiella*, *Pantoea*, *Serratia*, *Erwinia,* and *Yersinia*) possessed OmpA with highly similar amino acid residues (Figure 4A,B). Residues corresponding to OmpA G80 to P305 of *E. coli* ATCC 25922 were aligned for analysis by using ClustalW, gap open penalty 1, gap extention penalty 0.05. The similarity scores of OmpA amino acid sequences of Enterobacteriaceae compared to *E. coli* were 69–92%, indicating that the amino acid residues were highly conserved among Enterobacteriaceae. Excluding the Enterobacteriaceae family, other orders of the class Gammaproteobacteria (genera *Pasteurella*, *Gallibacteruim*, *Glaesserella*, *Bibersteina*, *Acinetobacter*, *Actinobacillus*, *Haemophillus*, *Vibrio,* and *Pseudomonas*) were less similar to *E. coli* in the OmpA sequence (Figure 4A). Of the Proteobacteria phyla, classes Betaproteobacteria (genera *Burkholderia* and *Bordetella*, Figure 4A) and Alphaproteobacteria (genus *Rickettsia*) were also less similar to *E. coli* in the OmpA sequence.

### 2.5. Induction of Extra-Intestinal Gland Inflammation by OmpA

**Figure 5:**
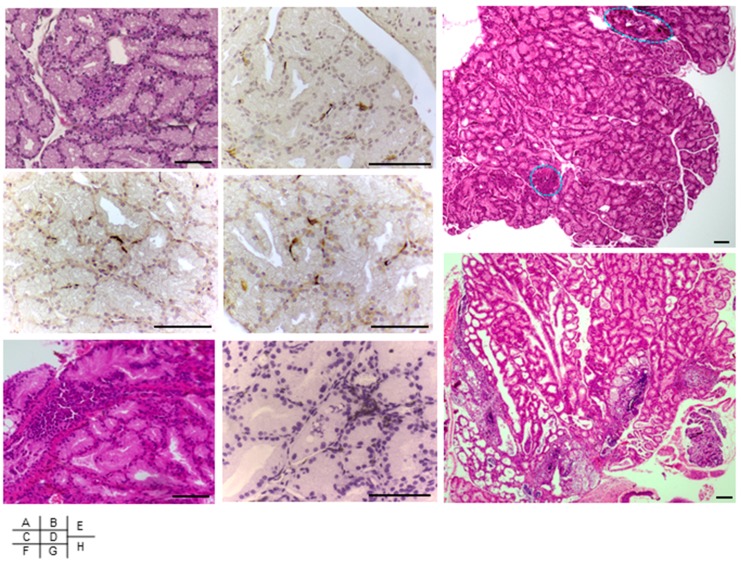

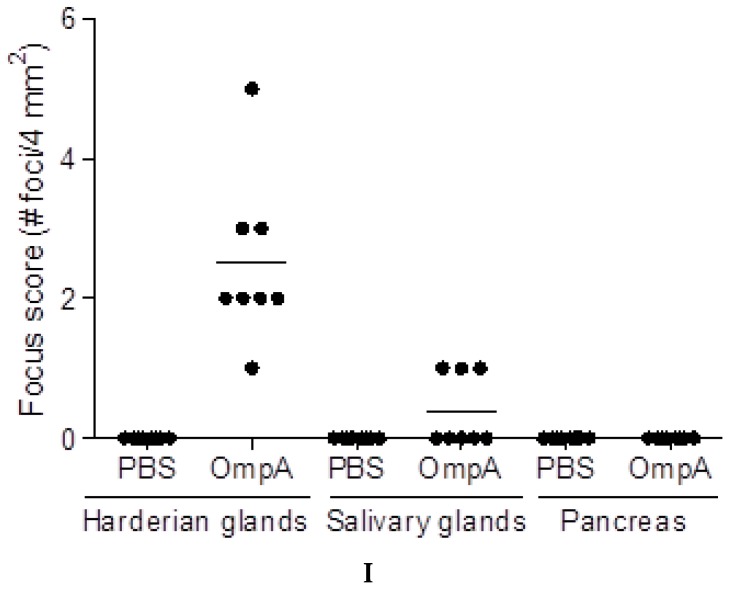
Histology of mice injected with OmpA. Harderian glands of C57BL/6 mice inoculated once a week during eight weeks with OmpA (**A**–**H**) were examined by HE-staining (**A**,**E**,**F**,**H**) and immune-staining for CD3 (**B**,**G**), CD4 (**C**), and CD8 (**D**), at fifteen weeks (**A**–**E**) or twenty-two weeks (**F**–**H**) after the final injection, in magnifications at 20× (**A**–**D**,**F**,**G**) and 4× (**E**,**H**). Dotted lines indicate inflammatory foci in a representative 4 mm^2^ of one mouse, fifteen weeks after the final inoculation of OmpA (**E**). Scale bar, 100 µm. Quantification of inflammatory foci in Harderian glands, salivary glands, and pancreas, fifteen weeks after the final inoculation of OmpA (**I**). Data presented are individual values (*n* = 8) plotted with a bar indicating the group mean. Differences between the groups of measurements were analyzed by Kruskal-Wallis with post-test comparison. Focus scores of the Harderian glands in OmpA-treated group were significantly higher (*p* < 0.05) compared to all other measurement groups (**I**).

OmpA of *E. coli* was cloned and expressed as an N-terminal 6×His-tagged recombinant protein by IPTG induction (Figure 3F, lanes 4 and 5). Purification of 6×His-OmpA (Figure 3F, lane 2) using Ni^2+^-affinity column resulted in elution of a single band, which reacted to OmpA–specific monoclonal antibody [28] (Figure 3F, lane 3). To investigate on the effect of the immunogenic OmpA protein, recombinant OmpA was injected to mice intraperitoneally once a week for eight weeks. Inoculation of the full length OmpA resulted in inflammatory cell infiltration of the Harderian glands at fifteen weeks (Figure 5A,E) and at twenty-two weeks (Figure 5F,H), after the final injection. Inflammation of the Harderian glands was observed in all eight mice per group, whereas no inflammatory cells were detected in those after inoculation of PBS (Figure 5I). Infiltrating cells in the Harderian glands of the OmpA-treated mice were in part CD3- (Figure 5B,G), CD4- (Figure 5C), and CD8- (Figure 5D) positive cells detected by immunohistochemistry, which were not detected in those of the PBS-treated mice. OmpA induced inflammation in the salivary glands in three of the eight mice per group, but not in the pancreas (Figure 5I).

### 2.6. Cytokine Production in Sera of Mice with Extra-Intestinal Inflammation Induced by OmpA

**Figure 6:**
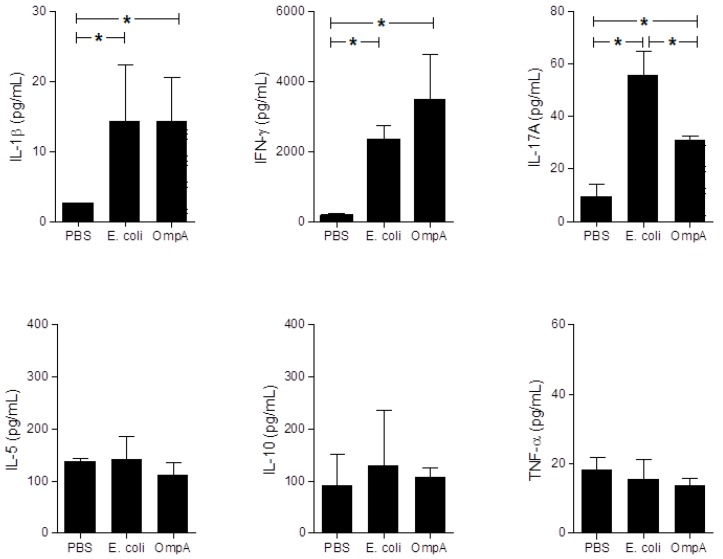
Cytokine production in mice injected with OmpA. Levels of IL-1β, IL-5, IL-10, IL-17A, IFN-γ, and TNF-α in sera of PBS (*n* = 8)-, *E. coli* (*n* = 8)-, and OmpA (*n* = 8)-treated mice were assessed using ELISA. Data presented as mean ± SEM analyzed by Kruskal-Wallis with post-test comparison. * *p* < 0.05.

Levels of cytokines in mice sera were measured, since systemic inflammatory responses may be able to trigger extra-intestinal gland inflammation. IFN-γ and IL-17A inflammatory cytokine levels and IL-1β innate inflammatory cytokine level were up-regulated in the sera of OmpA- and *E. coli*-treated mice, compared to those of the PBS-inoculated control mice. However, *E. coli* or OmpA administration did not affect IL-10, IL-5, and TNF-α production in sera (Figure 6).

### 2.7. Effect of Autoantibody Production by OmpA

**Figure 7:**
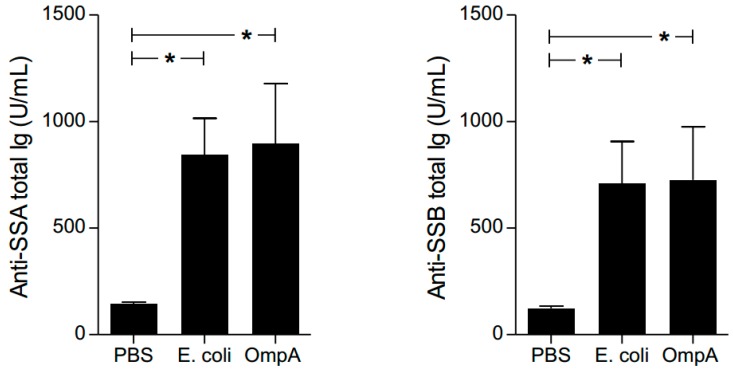
Autoantibody production by bacteria and bacteria-derived OmpA. Levels of anti- SSA/Ro and SSB/La antibodies in sera of PBS (*n* = 8)-, *E. coli* (*n* = 8)-, and OmpA (*n* = 8)-treated mice were assessed using ELISA. Data presented are mean ± SEM analyzed by Kruskal-Wallis with post-test comparison. * *p* < 0.05.

Anti-SSA/Ro and anti-SSB/La antibodies are autoantibodies elevated in sera of patients with systemic autoimmune diseases such as rheumatoid arthritis, lupus, and SS [30,31]. Thus, we examined whether OmpA-treated mice produced autoantibodies similar to those in autoimmune patients. Serum levels of anti-SSA/Ro antibodies and anti-SSB/La antibodies were elevated in OmpA- and *E. coli*-inoculated mice, compared to those inoculated with PBS (Figure 7). The data together with the histology of the inflammation in the extra-intestinal glands suggested that our model mouse had developed systemic autoimmune responses.

## 3. Discussion

The human gut harbors a highly complex microbial community that allows the digestion of diet and has profound influence on the immune health, hence dysbiosis has been implicated in the immune tolerance modulation or the development of autoimmunity. We previously reported on the significant role of a commensal bacterial strain of *E. coli* in the systemic immune activation and the generation of exocrinopathy in mice [32]. In the mice of the present study, the Harderian and salivary glands showed infiltrates of inflammatory cells by *E. coli*-treatment. Dysbiosis of the gut commensal bacteria have been reported to be associated with autoimmune disease severity [16,33,34,35]. In the present study, we found that repeated inoculation of OmpA from commensal *E. coli* could induce autoantibody production accompanied by inflammation of the Harderian and salivary glands in mice. Anti-SSA/Ro and anti-SSB/La antibodies have been reported to be elevated in sera of patients with autoimmune diseases upon exposure to intracellular autoantigens, which are discharged into the microenvironment through secretion of autoantigen-containing exosomes or by cell death [31]. The development of the pathologic responses in mice by OmpA may be due to chronic systemic immune activation and autoimmunity.

Microbe-derived structures have been reported to possess the ability to induce autoimmune diseases in experimental animals [36]. Chronic stimuli of the immune system may be provided by extra-intestinal translocation or overgrown microorganisms of a dysbiotic gut. To address the role of microbe-derived molecules in the patho-etiology of autoimmunity, normal mice were stimulated repeatedly with a broad range of pathogen-associated molecular patterns (PAMPs) (PG, TLR-2 ligand; LTA, TLR-2 ligand; MDP, NOD-2 ligand; LPS, TLR-4 ligand). Stimulation with PGN, MDP, LTA, or LPS did not induce infiltration of inflammatory cells in the Harderian or salivary glands. It may be possible that the molecules did not possess the combined functionality of immunogenic antigens and bystander adjuvants [37], which are indispensable for autoimmunity [38]. In addition to the microbial molecules investigated in the present study, we previously studied the flagellar filament structural protein FliC, a TLR5-ligand, and an NLR apoptosis inhibitory protein (NAIP) activator, derived from commensal *E. coli*, which was repeatedly inoculated following the same regimen of injection as the present study [17,39]. In the reports, we had found that sialadenitis and pancreatitis could be induced by FliC in mice within the fifteen weeks of observation after the final inoculation [17,39]. The results of the present study indicated that OmpA was highly antigenic to induce humoral adaptive immune response to elicit antibodies and to encounter histological inflammation of the Harderian and the salivary glands, but not of the pancreas, indicating that different microbe-derived proteins had initiated inflammation in exocrine glands with tropism.

In our previous study, pancreas of mice after *E. coli*-derived FliC treatment showed infiltration of inflammatory cells, and the majority of the infiltrated cells were CD3^+^ T cells [39]. In the present study, inflammation of the Harderian glands was reproduced in immune-deficient Rag2 KO mice and in mature T cell-deficient TCRβ-TCRδ KO mice by adoptive transfer of splenocytes or CD4^+^ T cells, respectively, from *E. coli*-treated mice. The results suggested that the exogenously administered *E. coli*-derived peptides possessed the ability to encounter cell-mediated immune response. Enterobacteriaceae, such as *E. coli* and *Klebsiella pneumoniae*, have the ability to use its cell surface OmpA to bind and to incorporate into dendritic cells (DCs) [40,41]. *Salmonella* OmpA induces expression of MHC class II and co-stimulatory molecules on DCs in mice [42] and differentiate naïve CD4^+^ T cell biased towards Th1 and Th17 [42,43,44,45]. In our mouse model, IL-1β, IFN-γ, and IL-17A were inflammatory cytokines that were predominantly produced in sera, similar to the cytokine production by FliC [17]. In addition to cytokine production, OmpA of *Klebsiella pneumoniae* after endocytosis by DCs has been shown to gain access to the cytosolic MHC class I presentation pathway to elicit cytotoxic T cells (CTLs), in the absence of CD4^+^ T cell help or adjuvant. This property of OmpA has been explained by its ability to bind scavenger receptors, such as LOX-1 [21,46], and favor antigen targeting to and cross-priming by DCs [40], which may explain the positivity of CD8^+^ T cells in the Harderian glands in OmpA-treated mice in the present study.

Although exact mechanisms remain unknown, shared epitopes between proteins of the host and the microbiome have been proposed in the initiation of autoimmune diseases. In autoimmune pathology, sequence similarity of skin, oral, and gut commensal bacteria with autoantigens of SS and lupus [16], and cross-reaction of envelope proteins in spondyloarthritis patients have been reported [47], which may in part explain the link between dysbiosis and the pathology of autoimmunity [14]. The Enterobacteriaceae family possesses OmpA with high post-transcriptional sequence similarities, indicating that the conserved sequence could be shed from a broad spectrum of microbiota. It may thus be speculated that not only *E. coli*, as in the present study, but multiple microorganisms belonging to the Enterobactericacea family may potentially trigger initiation of autoimmunity. Validation of sequence differences of OmpA in patient isolates will allow further elucidation of the role of OmpA in the pathogenesis of autoimmunity.

In conclusion, we found a novel antigen of the commensal bacteria with a possible association to the pathogenesis in autoimmunity in mice. The highly immunogenic OmpA may be responsible for *E. coli* to induce innate and Th1/Th17-associated pro-inflammatory cytokines and CTLs, which may contribute to the acinar cell destruction of exocrine glands. Since we were not able to elucidate the interaction of OmpA with the exocrine glands, future analysis is needed to determine responsible OmpA residues for autoantibody production. Peptide sequence differences may explain the inflammatory involvement in the pancreas by FliC, but not by OmpA, despite similar cytokine production by the two Enterobactericeae derivatives.

## 4. Materials and Methods

### 4.1. Bacterial Strains, Culture Conditions and Bacteria-Derived Components

A nonpathogenic human isolate *E. coli* strain ATCC 25922 (*bfpA*^−^, *eaeA*^−^, *st*^−^, *lt*^−^, *stx1*^−^, *stx2*^−^, *ial*^−^, *aggR*^−^, *daaE*^−^) was used for animal injection experiments [32]. Bacteria were cultured in Brain Heart Infusion broth (BD Pharmingen, Franklin Lakes, NJ, USA) aerobically for 18 h at 37 °C with vigorous shaking. Bacteria were then harvested, washed twice with PBS (pH 7.4), resuspended in PBS, and heated at 80 °C for 30 min. *E. coli* DH5α (TaKaRa Bio, Shiga, Japan) and BL21 (DE3) (New England Biolabs, Ipswich, MA, USA) were used to clone and to express recombinant proteins, respectively, and were cultured in Luria–Bertani (LB) medium (Invitrogen, Life Technologies, Carlsbad, CA, USA) incorporated with 50 µg/mL ampicillin (Sigma Chemicals, St. Louis, MO, USA). LPS from *E. coli*, PGN and LTA from *Staphylococcus aureus*, were purchased from Sigma Chemicals. MDP was purchased from Invitrogen.

### 4.2. Mice

C57BL/6 wild type mice and TCRβ-TCRδ knockout (KO) mice on C57BL/6 background were purchased from Sankyo Labo Service Corporation (Tokyo, Japan) and The Jackson Laboratories (Bar Harbor, ME, USA), respectively. Rag2 KO mice on C57BL/6 background were kindly provided by Professor Ryo Abe (Tokyo University of Science, Tokyo, Japan). Six weeks old female mice were used for the experiments. All experiments were performed in the animal facility at the Department of Microbiology and Immunology, Tokyo Women’s Medical University (TWMU), approved by the Ethics Review Committee for Animal Experiments, TWMU (project identification code AE18-061, approval date 30 March 2018).

### 4.3. Treatment of Mice with Bacteria and Bacterial Components

Mice were treated with or without bacteria or bacterial cell wall components. Heat-killed *E. coli* (2 × 10^7^ colony forming units), 10 µg of PGN, MDP, LTA, LPS or recombinant OmpA in 200 μL PBS, respectively, were injected intraperitoneally once a week for a total of 8 weeks. Each treatment group consisted of seven to eight mice. Mice were sacrificed to obtain samples subsequent to euthanasia with carbon dioxide at the indicated time after the final injection.

### 4.4. Isolation of Extra-Intestinal Gland Tissues

Harderian glands and submandibular salivary glands were removed following euthanasia. Tissue was fixed in 10% buffered formalin.

### 4.5. Sera Collection

Mice were bled by cardiac puncture after euthanasia. Samples were placed at room temperature for 2 h, centrifuged at 900× *g* for 20 min, and sera were collected and stored at −20 °C until use.

### 4.6. Adoptive Transfer Experiments

Single-cell suspensions from spleen of C57BL/6 wild type mice injected once a week during eight weeks with *E. coli*, OmpA, or PBS, were obtained at one week after the final inoculation. Bulk spleen cells, or splenic T cells separated using anti-mouse CD4 antibody by magnetic beads on AutoMACS (Miltenyi Boitec, Bergisch Gladbach, Germany) were transferred intravenously to Rag2 KO and TCRβ-TCRδ KO mice on C57BL/6 background, respectively. Each treatment group consisted of six mice. Mice were sacrificed one week after cell transfer to obtain tissue samples.

### 4.7. Histology

Tissue preparation was described previously in Reference [31]. Briefly, four micrometer-thick sections of Harderian and salivary glands were stained with HE. For immunohistochemical assessment, sections were deparaffinized, rehydrated, and then heated in 10 mM sodium citrate buffer (pH 6.0) for 20 min at 121 °C for antigen retrieval. Subsequently, sections were incubated with 0.3% H_2_O_2_ in methanol for inhibition of endogenous peroxidase for 30 min and incubated with primary antibodies diluted 1:30 in 1% bovine serum albumin in PBS (BSA-PBS) for 60 min at room temperature. Primary biotinylated antibodies included hamster anti-CD3ε (clone 145-2C11; BD Pharmingen), rat anti-CD4 (clone RM4-5; BD Pharmingen), rat anti-CD8α (clone 53-6.7, BD Pharmingen), which were detected by horse radish peroxidase (HRP)-conjugated streptavidin (Dako, Glostrop, Denmark), and rabbit anti-IgG1 (Novus Biologicals, Littleton, CO, USA), which was detected by EnVision FLEX (Dako). Immunoreactivity was visualized using 3,3′-diaminobenzidine substrate chromogen (Dako), counterstained with Mayer’s hematoxylin (Muto Pure Chemicals, Tokyo, Japan). The slides were examined on Biorevo BZ9000 microscopy (Keyence, Osaka, Japan) and Vanox ADH (Olympus, Tokyo, Japan).

### 4.8. Focus Scoring

Inflammation in the exocrine gland was quantified using focus scores, as previously described in Reference [48]. Inflammation of at least 50 mononuclear cells was considered as a focus, and the numbers of inflammatory foci per 4 mm^2^ were determined as focus scores. Digital measurement of the surface area of the sections and the cell counts were performed on BZ-II Analyzer version 2.00 (Keyence). Inflamed specimen was determined by the presence of more than one focus. Total focus score was calculated for each individual mouse.

### 4.9. Two Dimensional Polyacrylamide Gel Electrophoresis (2-D PAGE) of E. coli Cell Surface Proteins

Water solubilization of *E. coli* ATCC 25922 surface proteins was performed as previously described in Reference [39]. No DNA was detectable in the spectrophotometric analysis at 260 nm. Then, 2-D PAGE was performed with 200 mg of the surface protein extract on Ettan IPGphor3 IEF using Immobiline DryStrip pH 6-11 (GE Healthcare, Little Chalfont, Buckinghamshire, UK), and 10% acrylamide gels with molecular weight markers between 25–100 kDa (Nippon Gene, Tokyo, Japan). Spot detection and matching were performed using Multi Gauge Software on FLA5100 (Fuji Film, Tokyo, Japan).

### 4.10. Western Blotting of Immunoreactive E. coli Surface Proteins

Proteins resolved in 2-D PAGE were transferred to nitrocellulose membrane (Bio-Rad Laboratories, Hercules, CA, USA), which was blocked in TBS-TM buffer (10 mM Tris-HCl pH7.4, 0.9% NaCl, 0.05% Tween 20, 10% skimmed milk) and incubated with serum of a mouse two months after the eighth inoculation of *E. coli*, or serum of a mouse treated with PBS alone, diluted 1:2000 in TBS-TM. Immunoreactive spots were detected using an Immunostar enhanced chemiluminescence kit (Wako Pure Chemical Industries, Osaka, Japan).

### 4.11. Identification of Proteins Using MALDI-TOF-MS

MALDI-TOF mass spectrometry (AutoflexII; Bruker Daltonics) was used for protein identification, as previously described in Reference [39]. Briefly, spots from SYPRO-Ruby (Life Technologies) stained 2-D PAGE gel were excised on EXQuest (Bio-Rad), and digested with trypsin (Trypsin Gold, Mass Spectrometry Grade; Promega Corporation, Fitchburg, WI, USA). Mass spectra were calibrated using a peptide calibration standard mono (Bruker Daltonics) and ran in the positive ion reflector mode and in a mass-to-charge ratio (*m/z*) range of 600–4000 Da. Peptide mass fingerprint (Matrix Science) was conducted using the non-redundant NCBI database (US National Library of Medicine) with MASCOT search engine (Matrix Science), through BioTools 3.0 interface (Bruker Daltonics).

### 4.12. Recombinant OmpA Protein Purification and Anti-OmpA Antibody Measurement

DNA fragments of the *ompA* gene encoding for OmpA1-325, was amplified by polymerase chain reaction (PCR) using InFusion DNA polymerase (TaKaRa Bio) and genomic DNA of *E. coli* ATCC 25922 as template. PCR products were inserted into TAGZyme pQE2 (Qiagen, Hilden, Germany) at *Sph*I and *Hind*III sites (New England Biolabs) in *E. coli* DH5α, which were selected with 50 µg/mL ampicillin in LB medium. After IPTG induction of *E. coli* BL21 transformed with constructed plasmids, cells were lysed in the presence of EDTA-free protease inhibitor cocktail (Sigma Chemicals). Then, 6×His-tagged recombinant OmpA was purified by Ni^2+^-affinity chromatography (GE Healthcare). Protein purity was assessed by SDS-PAGE stained with Coomassie brilliant blue and Western blotting probed with anti-OmpA monoclonal antibody 49.415 [30]. After treatment with Detoxin Gel Endotoxin Removing Columns (TaKaRa Bio), endotoxin levels in the recombinant protein solution used for experiments were <0.005 EU/ml measured by using the Toxin Sensor Chromogenic Limulus Amebocyte Lysate Endotoxin Assay Kit (GenScript, Piscataway, NJ, USA). Protein concentration was quantified with the bicinchoninic acid (BCA) assay (BioRad). Anti-OmpA antibody levels in sera were measured by detection with HRP-conjugated goat anti-mouse antibodies (BioSource, Camarillo, CA, USA) against purified recombinant OmpA on nitrocellulose membrane strips, followed by determination of the intensities using the ImageJ program/gel analyzer option (US National Institute of Health, Bethesda, MD, USA), as previously described in Reference [32].

### 4.13. Bioinformatic Analysis of the OmpA Protein

OmpA amino acid sequences from representative environmental and pathogenic strains of the Proteobacteria phyla deposited to NCBI (US National Library of Medicine) were subjected to phylogenetic analysis by using the Maximum Likelihood method (MEGA X software, Molecular Evolutionary Genetics Analysis) [29]. Gammaproteobacteria/Enterobacteriales/Enterobactericeae genera *Escherichia*, *Salmonella*, *Citrobacter*, *Cronobacter*, *Enterobacter*, *Klebsiella*, *Serratia*, *Pantoea*, *Erwinia,* and *Yesinia*; Gammaproteobacteria/Pasturellales/Pasturellaceae genera *Pasteurella*, *Gallibacterium*, *Glaesserella*, *Bibersteinia*, *Acinobacillus,* and *Haemophilus*; Gammaproteobacteria/Pseudomonadales genera *Pseudomonas* and *Acinetobacter*; Gammaproteobacteria/Vibrionales genus *Vibrio*; Betaproteobacteria/Burkholeriales genera *Burkholeriales* and *Bordetella*; Alphaproteobacteria/Rickettsiales genera *Anaplasma* and *Rickettsia* were included in the phylogenic analysis. OmpA amino acid sequences of Gammaproteobacteria/Enterobacteriales/Enterobactericeae: *Escherichia coli* (ATCC 25922), *Salmonella enterica* subsp. enterica serovar Typhimurium (GenBank AIH09359.1), *Citrobacter freundii* (GenBank AWW73248.1), *Cronobacter sakazakii* (GenBank AAY18798.1), *Enterobacter cloacae* (GenBankAAW 73250.1), *Klebsiella pneumoniae* (GenBank CCI75899.1), *Serratia marcescens* (GenBank ALE95511.1), and *Yesinia pestis* (GenBank AAM86287.1) were subjected to multiple sequence alignment (ClustalW, available online: https://www.genome.jp/tools-bin/clustalw).

### 4.14. Enzyme-Linked Immunosorbent Assay (ELISA)

Serum levels of anti-SSA/Ro and anti-SSB/La antibodies (Alpha Diagnostics International, San Antonio, TX, USA) were determined using ELISA kits according to the manufacturer’s protocol. Serum inflammatory cytokines in mice sera were measured by sandwich-ELISA using capture antibodies against IL-1β (clone B122; eBioscience, Affymetrix Japan, Tokyo, Japan), IL-5 (clone TRFK5; eBioscience), IL-10 (clone JESS-16E3; eBioscience), IL-17A (clone 17CK15A5; eBioscience), IFN-γ (clone XMG1.2; eBioscience), TNF-α (clone TN3-19.12; BD Pharmingen), and biotinylated-detection antibodies against IL-1β (clone 13-7112-85; eBioscience), IL-5 (clone TRFK4; eBioscience), IL-10 (clone JESS-2A5; eBioscience), IL-17A (clone 17B7; eBoiscience), IFN-γ (clone R4-6A2; eBioscience), TNF-α (clone MP6-XT22; BD Pharmingen). All antibodies were diluted 1:1000 in PBS containing 10% fetal calf serum (Gibco, Thermo Fisher Scientific, Tokyo, Japan). Biotinated antibodies were detected by HRP-conjugated avidin (Vector laboratories, Burlingame, CA, USA) and visualized by 3,3′,5,5′-tetramethylbenzidine (Dako), where the reaction was stopped with 2 M H_2_O_2_. Microtitier plate reader (Vmax, Molecular Devices, Tokyo, Japan) at 450 nm was used to measure the optical densities.

### 4.15. Statistical Analysis

Multiple comparisons of the data were performed using Kruskal-Wallis with a post-test comparing each group to all other groups on GraphPad Prism version 5 for Windows (GraphPad Software, San Diego, CA, USA). *p*-values < 0.05 were considered as statistically significant.
